# Medication-related osteonecrosis of the jaw in multiple myeloma patients: a case series

**DOI:** 10.1038/s41415-026-9628-4

**Published:** 2026-07-10

**Authors:** Louis Madden, Dermot Pierse

**Affiliations:** 244671085177307194569 Division of Oral and Maxillofacial Surgery, Oral Medicine, Oral Pathology and Oral Radiology, Dublin Dental University Hospital, Trinity College, Ireland; Department of Oral and Maxillofacial Surgery, University Hospital Galway, Galway, Ireland; 308805696804008586114 Division of Oral and Maxillofacial Surgery, Oral Medicine, Oral Pathology and Oral Radiology, Dublin Dental University Hospital, Trinity College, Ireland

## Abstract

Medication-related osteonecrosis of the jaw (MRONJ) is a complication of antiresorptive and anti-angiogenic therapies used in the management of malignancy and can cause significant functional and aesthetic morbidity for patients. Multiple myeloma (MM) is a plasma-cell haematological malignancy frequently associated with osteolytic bone disease. Antiresorptive therapy (ART) reduces skeletal-related events in MM and other malignancies with skeletal involvement but is associated with an increased risk of MRONJ. This paper presents the clinical features and management of four MRONJ cases, ranging from localised exposed bone to severe maxillary and mandibular destruction, in MM patients who presented to the Dublin Dental University Hospital between 2021 and 2023. MRONJ management is challenging due to its refractory nature. As prevention is paramount, dental practitioners play a key role in minimising risk through comprehensive dental assessments and management before, during and after ART. Multidisciplinary collaboration between dental practitioners and oncology teams, alongside timely diagnosis and risk reduction, is essential to improve outcomes and minimise MRONJ-related complications. These cases are contextualised within current evidence on MRONJ in malignancy, focusing on implicated medications, risk factors, prognostic factors and treatment outcomes.

## Key points

- Multiple myeloma patients are at high risk of medication-related osteonecrosis of the jaw (MRONJ) because antiresorptive therapy is frequently required to prevent skeletal-related events, often with prolonged or sequential exposure to bisphosphonates and denosumab.
- The four cases illustrate severe MRONJ in multiple myeloma after prolonged antiresorptive therapy, with advanced complications including oro-nasal communication and mandibular pathological fracture. The literature supports cumulative antiresorptive exposure, dental infection, extractions, periodontal disease and denture trauma as important risk factors.
- Pre-ART dental assessment, infection control and regular dental review are essential. Exposed bone, sinus tracts or non-healing extraction sockets should prompt early referral to oral and maxillofacial surgery.

## Introduction

Medication-related osteonecrosis of the jaw (MRONJ) is defined as exposed bone, or bone probed through an intra-oral or extra-oral fistula in the maxillofacial region, persisting for over eight weeks without prior radiation or metastatic jaw disease.^1^ The incidence and severity of MRONJ are influenced by the type, potency and cumulative exposure to anti-resorptive therapies (ART), particularly intravenous bisphosphonates and denosumab.^2^^,^^3^ The alveolar bone is especially vulnerable due to high bone turnover and ART-induced inhibition of osteoclast activity and bone remodelling, particularly following dental extractions.^4^

Clinical presentations range from asymptomatic exposed bone to severe pain, infection, swelling, oro-antral/oro-nasal communications and pathological fractures.^1^ As management can be complex with refractory disease, guidelines from the American Association of Oral and Maxillofacial Surgeons (AAOMS), the Scottish Dental Clinical Effectiveness Programme and the Royal College of Surgeons of England emphasise prevention and early detection.^1^^,^^5^^,^^6^ While MRONJ occurs across various malignancies, it is especially prevalent in multiple myeloma (MM) patients.^7^

MM, usually diagnosed in a person's late sixties, is a heterogeneous plasma cell malignancy with a rising global incidence.^8^^,^^9^^,^^10^^,^^11^ Diagnosis is based on the CRAB criteria: hypercalcaemia (Calcium elevation), Renal insufficiency, Anaemia and Bone lesions, along with biomarker-based myeloma-defining events (≥60% bone marrow plasma cells, serum free light chain ratio ≥100, or ≥1 focal lesion on MRI).^12^^,^^13^ Osteolytic bone disease affects up to 80% of MM patients and significantly contributes to morbidity, with fractures occurring in 50% of cases.^14^

MM treatment commonly involves proteasome inhibitors, immunomodulatory agents and corticosteroids.^15^ Furthermore, guidelines recommend ART for all active MM patients to prevent osteolytic lesions and skeletal-related events (SREs).^16^^,^^17^ Despite improving survival and quality of life, these therapies may predispose patients to MRONJ through their osteoclast inhibition and anti-angiogenic effects.^18^^,^^19^^,^^20^

This paper presents four MRONJ cases in MM patients treated at the Dublin Dental University Hospital with a narrative overview of the literature on MRONJ in malignancy.

## Case series

### Case 1

A patient in their seventies presented in February 2022 with a four-month history of right-sided facial pain, gingival swelling, halitosis and spontaneous loss of five maxillary teeth over six weeks. In October 2021, a PET scan with haematology excluded active extra-medullary MM and a gingival biopsy performed by the ear, nose and throat team showed pseudoepitheliomatous gingival hyperplasia without evidence of dysplasia.

The patient was diagnosed with IgG lambda MM with laryngeal involvement in 2016 and received two cycles of RVD (bortezomib, lenalidomide, dexamethasone) followed by an autologous stem cell transplantation (ASCT) in 2017, achieving a very good partial response. At presentation, MM was in remission and the patient was taking maintenance lenalidomide (21/28 days). The patient received monthly intravenous zoledronic acid (ZA) (4 mg) from January 2017 until March 2020, then switched to monthly denosumab (120 mg) until August 2021, when it was ceased due to oral symptoms. Medical history was also significant for hypertension, hypercholesterolaemia and hypothyroidism. They did not smoke or consume alcohol.

**Fig. 1:**
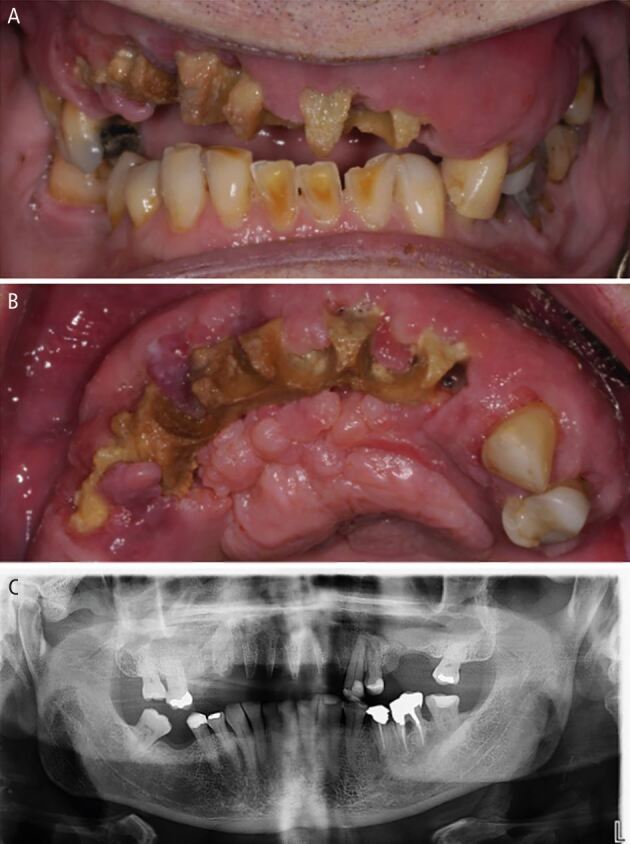
Case 1. (A, B) Initial clinical presentation. (C) Initial orthopantogram presentation

**Fig. 2:**
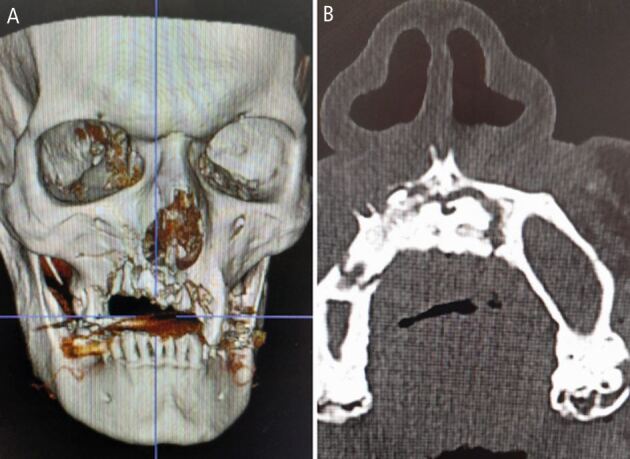
Case 1. (A) CT imaging 3D reconstruction. (B) CT axial view showing extensive maxillary osteonecrosis with a moth-eaten appearance, significant resorption of the alveolar process resulting in loss of most anterior teeth, extension into the palatine process, involvement of both maxillary sinus walls and multiple cortical defects with sequestered bone foci

Pre-existing oral health findings included anterior tooth surface loss, periodontal disease and a heavily restored dentition, with no history of denture use. Clinical examination revealed suppuration on palpation of the granulomatous, erythematous and hypertrophic gingiva of the anterior hard palate with exposed bone extending from 16–23 (Fig. 1). Computed tomography (CT) confirmed MRONJ (Fig. 2).

Sharp bony spicules were removed for comfort. Exposed bone was irrigated with 0.2% chlorhexidine. The patient was prescribed metronidazole 400 mg and amoxicillin 500 mg three times daily (TDS) for five days, followed by doxycycline 100 mg once daily (OD) for 14 days, along with 0.2% chlorhexidine mouthwash twice daily (BD).

**Fig. 3:**
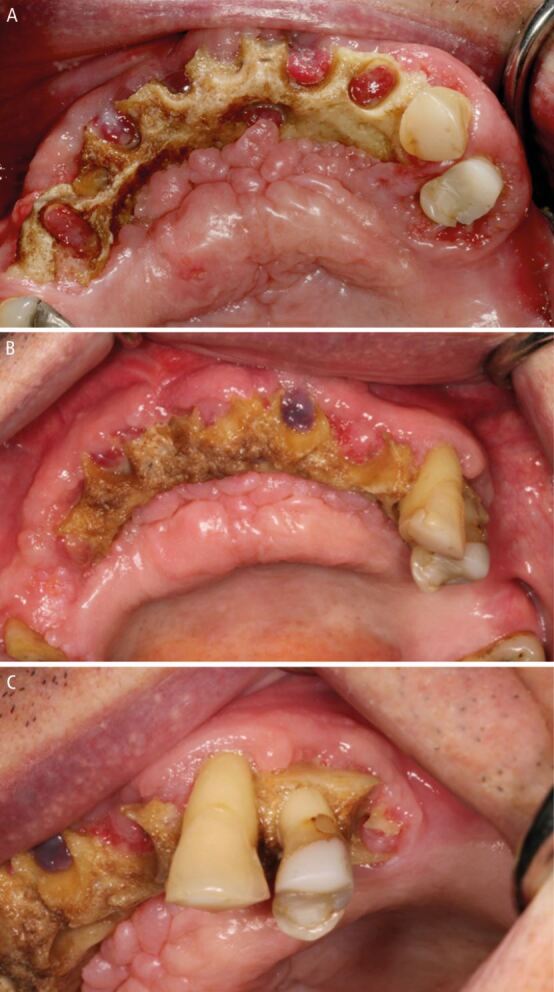
Case 1. (A) Clinical presentation at one-month review. (B, C) Clinical presentation at three-month review

By March 2022, following completion of doxycycline 100 mg OD for 14 days, the patient reported an improvement in halitosis and resolution of pain. Although there was an improved gingival appearance, the large area of exposed bone persisted. Sharp bony edges were smoothed and further doxycycline 100 mg OD for 14 days was prescribed (Fig. 3).

**Fig. 4:**
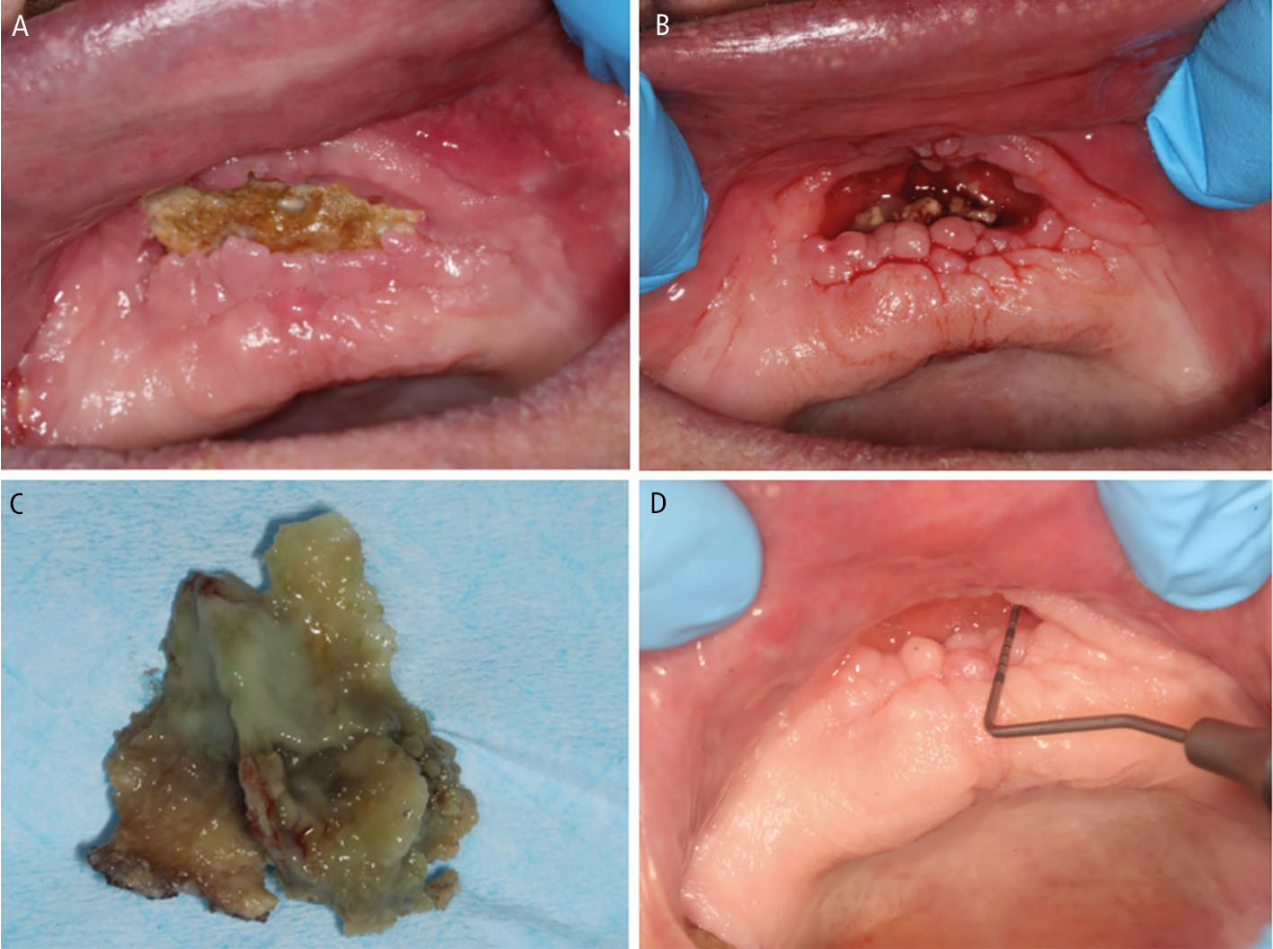
Case 1. (A) Reduced area of MRONJ at review post-surgical debridement. (B) Anterior maxilla immediately following sequestrectomy. (C) Sequestrum. (D) Full mucosal coverage at review post sequestrectomy

Under general anaesthetic (GA), necrotic bone was extensively debrided to healthy bleeding bone and primary closure was achieved with 3-0 Vicryl-Rapide sutures. By November 2022, the patient reported significant improvement, with reduced bone exposure in the 11–23 region (Fig. 4A). Due to the high surgical risk of oro-nasal communication, a conservative approach was taken to allow natural sequestrum exfoliation. At review, the sequestrum, involving the nasal cavity base, had become mobile and was removed (Fig. 4B, 4C). Although complete mucosal epithelialisation was observed, a pinpoint oro-nasal communication persisted and was managed conservatively with an obturator denture (Fig. 4D).

### Case 2

A patient in their eighties was referred in November 2021 for assessment of asymptomatic exposed bone in the anterior maxilla, persisting six months after extraction of a periodontally involved 14.

The patient was diagnosed with IgA Lambda MM in 2011. Initial MM treatment involved eight cycles of VMP (bortezomib, melphalan, prednisone). After relapse, they received 13 cycles of elotuzumab, lenalidomide and dexamethasone in 2012, followed by 20 cycles of pomalidomide, bortezomib and dexamethasone (2015-2019). At presentation, they had been receiving daratumumab, bortezomib and prednisone for refractory MM since October 2020, along with bi-monthly intravenous immunoglobulin for treatment-induced hypogammaglobulinaemia. ART history included monthly denosumab (60 mg) from October 2020 until onset of oral symptoms in October 2021. Anamnesis also included hypertension and gastro-oesophageal reflux disease. They did not smoke or consume alcohol.

**Fig. 5:**
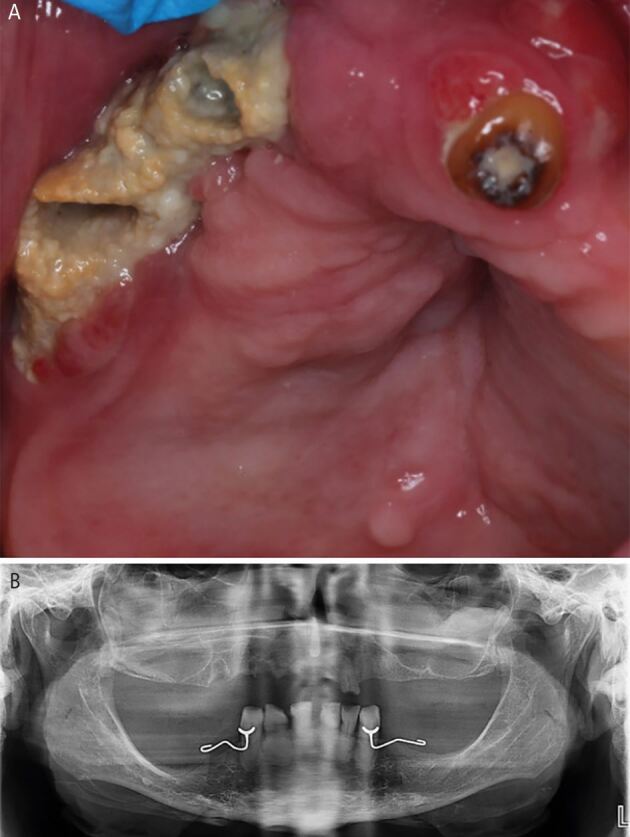
Case 2. (A) Initial presentation. (B) Initial orthopantogram presentation

**Fig. 6:**
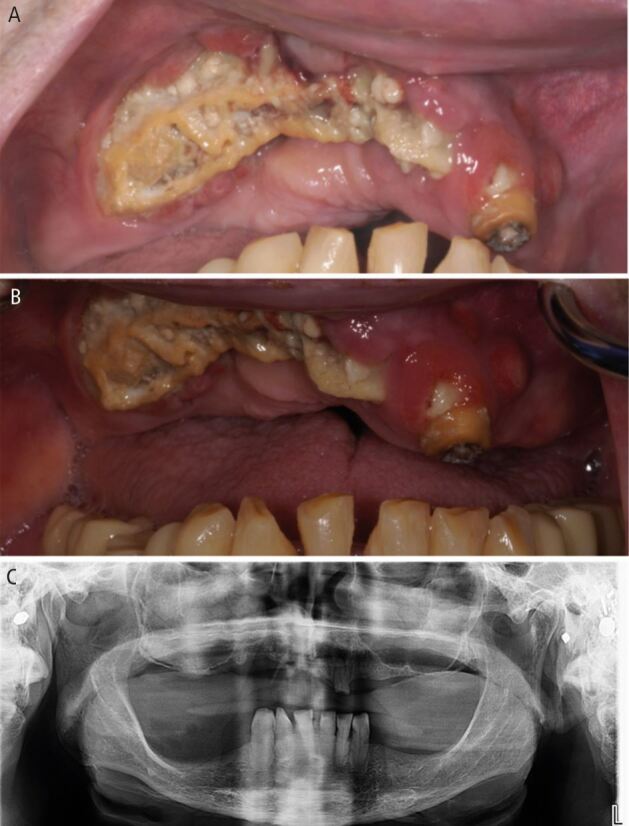
Case 2. (A, B) Clinical presentation at three-month review. (C) Orthopantogram at three-month review

Pre-existing oral health findings included periodontal disease and a history of maxillary and mandibular denture use. Clinically, a large area of necrotic bone with surrounding erythematous gingiva extended from 17–22 region (Fig. 5). There was suppuration on palpation of the labial gingiva of a retained 23 root. Sharp bone edges were removed using bone rongeurs. The patient was prescribed doxycycline 100 mg OD for 28 days and instructed to use 0.2% chlorhexidine BD. At one-month review, bone exposure had progressed, extending from the 16–24 site, with inflamed, erythematous gingiva around 23. Conservative debridement of loose necrotic bone and saline irrigation were performed, and doxycycline 100 mg OD was prescribed for one month. By May 2022, persistent exposed bone, suppuration and halitosis prompted surgical intervention (Fig. 6).

**Fig. 7:**
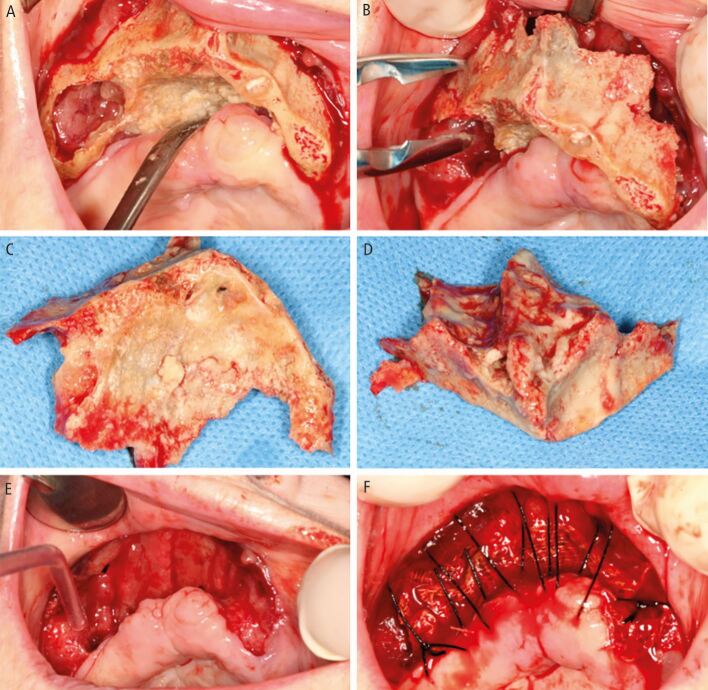
Case 2. (A, B, E, F) Intra-operative photographs of surgical debridement. (C, D) Necrotic bone specimen removed during debridement

**Fig. 8:**
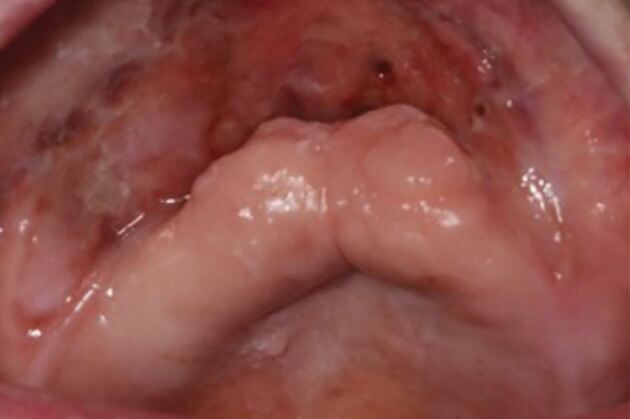
Case 2: post-surgical debridement review showing full healing and mucosal closure

Extensive debridement of necrotic bone was performed under GA, although limited to prevent an oro-nasal communication. The right maxillary sinus was exposed, irrigated with saline and packed with iodine-soaked oxidised cellulose. Concurrent extraction of 23 was performed. One-month post-operatively, no improvement in the MRONJ prompted further surgical debridement (Fig. 7). Intra-operatively, the entire exposed segment of the anterior and right maxilla had become mobile from the base and was deemed non-viable. The segment was removed, exposing the soft tissue of the nasal floor and right antral lining. A small oro-nasal fenestration was identified, adjacent soft tissues were advanced and closed over the fenestration using 6-0 Vicryl-Rapide sutures and a Whitehead's varnish pack was secured over the site with continuous 3-0 silk sutures. Post-operatively, amoxicillin 500 mg TDS for five days was prescribed. One-month post-operatively, the Whitehead's varnish pack was removed, revealing complete mucosal healing with no oro-nasal communication (Fig. 8).

### Case 3

A patient in their sixties was referred in September 2023 following a one-year history of persistent right mandibular pain, progressively worsening over the previous six months. Three weeks before presentation, the patient developed a numbness of the lower right lip. Notably, the 45 had been extracted six months earlier with no improvement in symptoms.

The patient had light chain MM diagnosed in 2019, treated with six cycles of RVD and an ASCT in 2020. At presentation, they were taking maintenance lenalidomide (21/28 days) and had received monthly intravenous ZA (4 mg) from October 2019 to March 2023. Comorbidities included osteoarthritis, irritable bowel syndrome and a hiatus hernia. The patient had no tobacco or alcohol history.

**Fig. 9:**
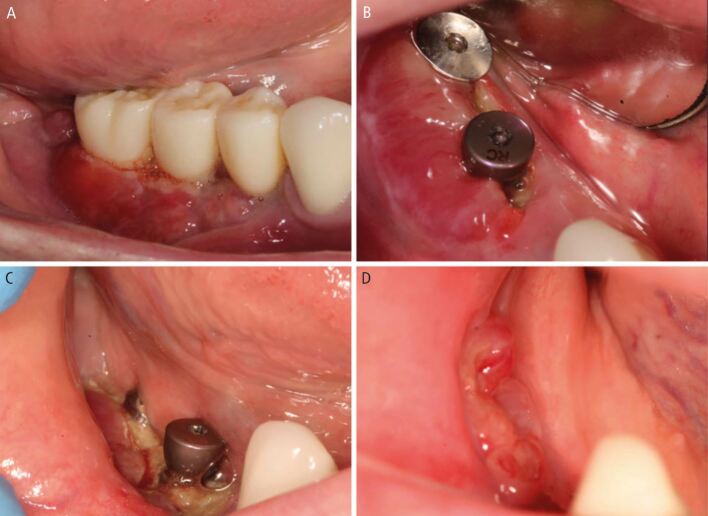
Case 3: (A) Initial clinical presentation. (B) Clinical presentation following removal of implant crowns. (C) Clinical presentation prior to surgical debridement. (D) Clinical presentation at review following first MRONJ debridement and implant removal

**Fig. 10:**
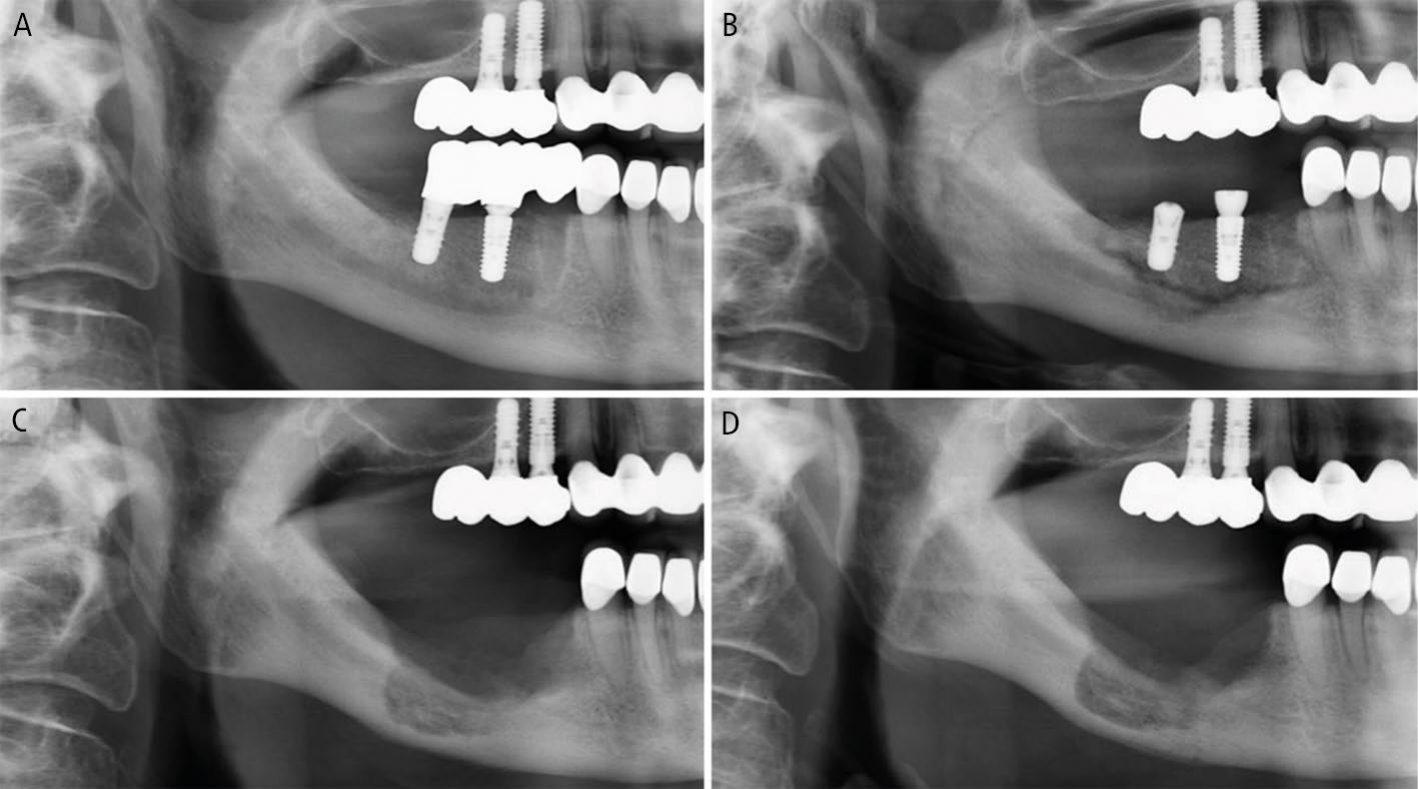
Case 3: (A, B, C, D) Orthopantogram of MRONJ progression from initial presentation to pathological fracture of right mandible

Baseline oral health assessment noted a heavily restored dentition with extracoronal restorations across the dentition and multiple implants, with no history of denture use. Extra-oral examination revealed right submandibular tenderness and altered sensation of the right mandibular branch of the trigeminal nerve. Intra-orally, an implant-supported bridge (45–46) surrounded by a suppurating soft, erythematous swelling was noted (Fig. 9A). Radiography confirmed a diagnosis of MRONJ with right mental nerve paraesthesia (Fig. 10).

A biopsy of erythematous soft tissue adjacent to the implant bridge confirmed an inflammatory process, excluding malignancy. The patient was prescribed Augmentin 625 mg TDS for seven days and 0.2% chlorhexidine mouthwash BD. While pain resolved after two weeks, paraesthesia persisted with exposed bone noted buccal and lingual to the 45–46 implants (Fig. 9B). Doxycycline 100 mg OD was commenced for 28 days.

Following six asymptomatic months, the patient presented with severe right mandibular pain and infected MRONJ around the 45–46 implants. Conservative treatment included metronidazole 400 mg and amoxicillin 500 mg TDS for five days. Progressive MRONJ necessitated surgical debridement (Fig. 9C). During the debridement, irrigation was performed with 0.2% chlorhexidine, povidone-iodine (1:10 saline dilution) and saline. Primary closure was achieved with 3-0 Vicryl-Rapide sutures and a Whitehead's varnish pack was secured with 3-0 silk. Post-operative antibiotics included metronidazole 400 mg and amoxicillin 500 mg TDS for five days.

**Fig. 11:**
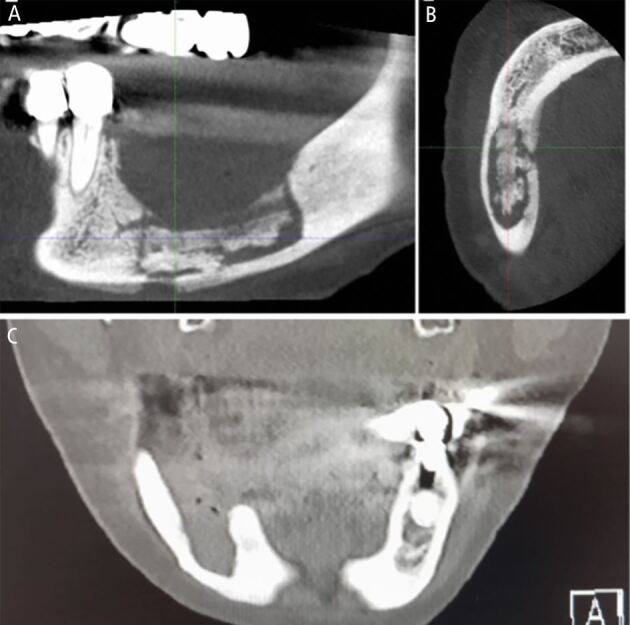
Case 3. (A) Pre-operative CBCT sagittal view of MRONJ. (B) Pre-operative CBCT axial view of MRONJ. (C) Most recent post-operative coronal CT imaging

Although initial pain relief was significant with no exposed bone noted at review (Fig. 9D), multiple intra-oral sinus tracts subsequently developed. This prompted further local debridement under local anaesthetic (LA), with removal of necrotic bone and granulation tissue, sparing the inferior mandible. A new Whitehead's varnish pack was applied and secured. Despite surgical intervention, MRONJ progression resulted in a non-displaced pathological fracture of the right mandible (Fig. 10D), which was managed conservatively with augmentin 625 mg TDS for ten days. The patient subsequently developed an orocutaneous fistula, which was drained under LA and irrigated with 10 mg gentamicin. The cavity in the lower right mandible was irrigated with iodine (1:10 saline dilution) and treated with 70 mg gentamicin. Vancomycin paste was placed intra-operatively and primary closure was obtained with 3-0 Vicryl-Rapide sutures. At review, fistula resolution and a bony callus was noted along the inferior mandibular border with CT reporting no evidence of further MRONJ progression (Fig. 11).

### Case 4

A patient in their sixties was referred in January 2021 following a three-month history of painless gingival swelling in the left anterior palate and significant weight loss. Before referral, haematology excluded a MM relapse. CT and blood testing showed MM in complete remission with normal serum free light chains and no obvious paraproteins.

The patient's MM was treated with four cycles of RVD and an ASCT in 2019. They received monthly intravenous ZA (4 mg) from October 2019 until the development of oral symptoms in November 2020. At presentation, they were taking maintenance 5 mg lenalidomide (14/28 days). Anamnesis also included hypertension and thrombocytopenia. The patient did not smoke or consume alcohol.

**Fig. 12:**
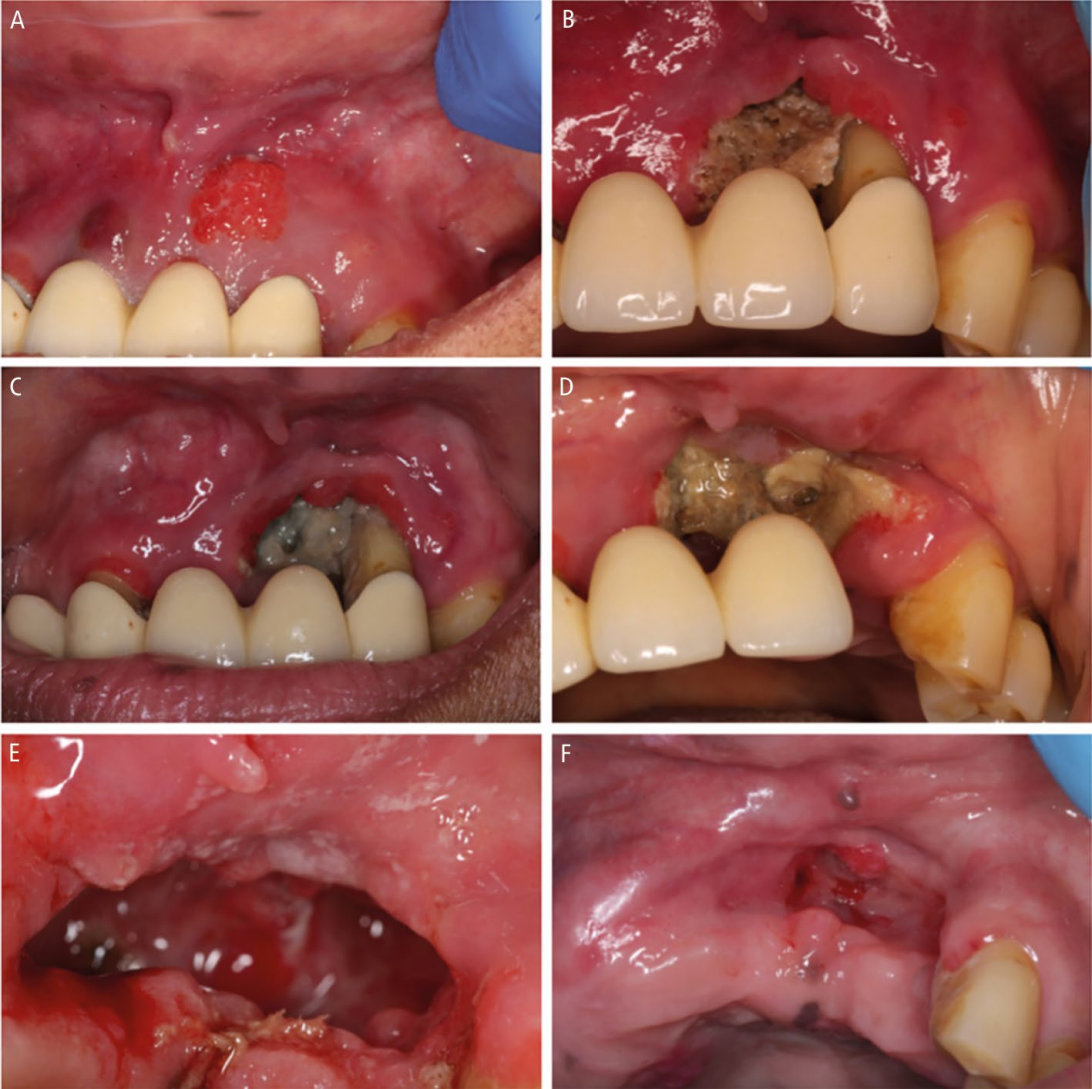
Case 4. (A) Initial clinical presentation. (B) Clinical presentation at three-month review. (C) Clinical presentation before first surgical debridement. (D) Review post-surgical debridement. (E) Review following removal of the upper left lateral incisor and bridge. (F) Full mucosal coverage and healing

**Fig. 13:**
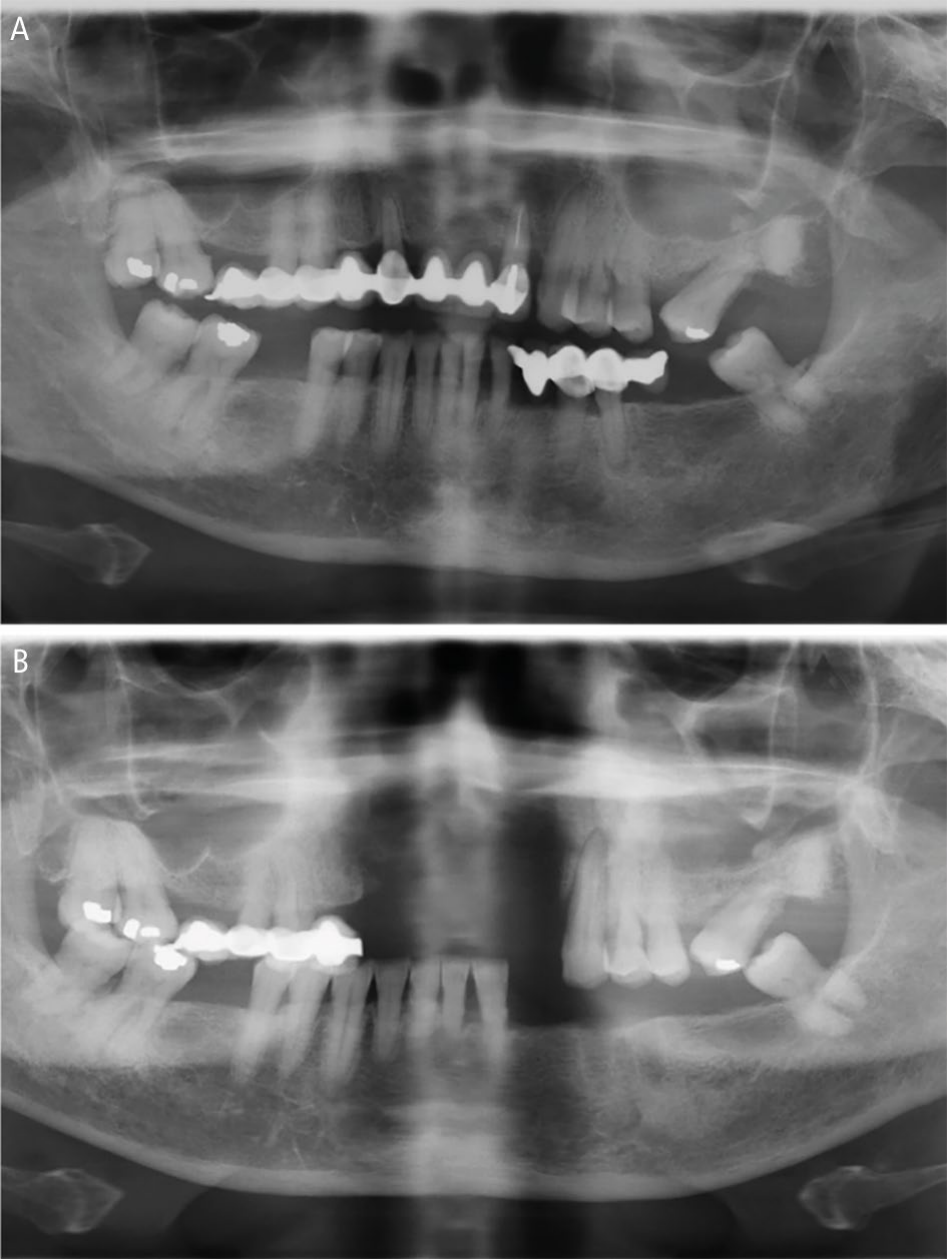
Case 4. (A) Initial orthopantogram presentation. (B) Final orthopantogram presentation

Pre-existing oral health findings included a heavily restored dentition with multiple fixed maxillary and mandibular bridges and periodontal disease, with no history of denture use. Clinical examination showed a fixed prosthesis from 16–22 and an erythematous buccal sinus tract adjacent to 22, where probing depths were 10 mm (Fig. 12A). A fluctuant, non-tender swelling was present in the left anterior palatal gingiva. An orthopantogram (OPG) was obtained (Fig. 13A).

A soft tissue biopsy of a sinus tract under LA, with pre-operative platelet infusion, revealed inflammatory granulation tissue, excluding malignancy. Post-biopsy, exposed anterior maxillary bone, a suppurating sinus tract and palatal swelling were noted. Initial treatment included metronidazole 400 mg and amoxicillin 500 mg TDS for five days, followed by doxycycline 100 mg OD for 14 days, with 0.2% chlorhexidine mouthwash BD.

At three-month review, although asymptomatic, the patient reported occasional nasal regurgitation of fluid when rinsing, managed with an obturator. Examination revealed increasing bone exposure, erythematous gingiva and suppuration (Fig. 12B). Exposed bone was copiously irrigated with saline and metronidazole 400 mg and amoxicillin 500 mg TDS were prescribed for five days. Following gingival improvement, doxycycline 100 mg OD was prescribed for three months.

Surgical debridement of MRONJ under LA to healthy bleeding bone was conducted with leukocyte platelet-rich fibrin (L-PRF) placement and primary closure with 5-0 Vicryl-Rapide sutures (Fig. 12C). Although initial healing and mucosal coverage was observed, three months later recurrence of bone exposure necessitated further surgical debridement, though the patient declined extensive debridement, despite likely full premaxillary involvement (Fig. 12D). Under LA, the 22 and bridge were extracted and exposed bone was debrided with placement of L-PRF and closure with 3-0 Vicryl-Rapide sutures. Post-operatively, doxycycline 100 mg OD for 28 days was prescribed. Following this post-operative course, bone exposure subsequently improved (Fig. 12E); the cavity was irrigated and iodine-dipped oxidised cellulose was placed in the cavity. Follow-up revealed asymptomatic and reduced bone exposure.

Due to persistent exposed bone, a final debridement of necrotic bone was carried out under LA; although, complete removal was not possible due to proximity to the nasal cavity and sinus. The area was steeped in topical gentamicin 0.1% for five minutes, L-PRF was placed and covered with a resorbable collagen membrane and sutured with 4-0 Vicryl-Rapide sutures. Post-operatively, augmentin 625 mg TDS for five days was prescribed. At review, sequestrectomy of the mobile segment of anterior maxillary MRONJ exposed the nasal floor which exhibited full mucosal coverage (Fig. 12F and Fig. 13B). The patient remained asymptomatic.

## Discussion

MRONJ occurs more frequently in MM, with reported incidences of approximately 5%, exceeding those reported in breast or prostate cancer cohorts.^7^ The Multinational Association of Supportive Care in Cancer/International Society of Oral Oncology (MASCC/ISOO) and American Society of Clinical Oncology (ASCO) guideline identifies cancer patients receiving oncologic-dose bone-modifying agents as the highest-risk group, with risk increasing with intravenous administration, cumulative exposure and sequential ART.^2^^,^^3^^,^^21^^,^^22^^,^^23^ In this series, all four patients received prolonged ART for MM-related bone disease (intravenous ZA/denosumab), including one with sequential bisphosphonate-denosumab exposure. While denosumab and ZA have generally been regarded as conferring comparable risk, more recent studies in oncology cohorts suggest that denosumab (120 mg monthly) may be associated with an earlier onset of MRONJ and, in some cohorts, a two- to three-fold higher incidence than ZA (4 mg monthly).^1^^,^^24^^,^^25^^,^^26^

Adjunctive antineoplastic agents, including anti-angiogenic agents and tyrosine kinase inhibitors, have been reported to cause MRONJ in patients with no history of ART use.^27^^,^^28^^,^^29^^,^^30^^,^^31^^,^^32^^,^^33^^,^^34^^,^^35^ This risk appears higher when these agents are used in combination with ART. Methotrexate has also been associated with MRONJ, including several cases occurring without prior ART.^36^^,^^37^^,^^38^ Accordingly, assessment of suspected MRONJ should include documentation of adjunctive antineoplastic agents, cumulative exposure and concurrent systemic therapies, as these may influence risk stratification and multidisciplinary management.

Chemotherapy, immunotherapy and corticosteroids are the most frequently reported medical risk factors for MRONJ.^39^^,^^40^^,^^41^^,^^42^^,^^43^^,^^44^ These agents may impair immunity, bone turnover and wound healing, increasing susceptibility to MRONJ.^40^^,^^45^^,^^46^ In a study of MM patients, thalidomide used with ZA for more than two years was associated with a 2.4-fold increased MRONJ risk.^47^ Other systemic and lifestyle-related factors contribute to MRONJ risk. While in this series, no patients had diabetes or a smoking history, diabetes has been associated with increased MRONJ risk, potentially due to delayed wound healing, microvascular ischaemia and impaired bone remodelling.^2^^,^^26^^,^^48^^,^^49^^,^^50^ Some studies also report up to a ninefold increased MRONJ risk among smokers, while others find no association.^2^^,^^41^^,^^45^^,^^51^^,^^52^ Despite conflicting evidence, reduced blood flow, impaired healing and smoking-related periodontal disease provide a plausible contributory mechanism.^51^

Although MRONJ most commonly affects the mandible (~75%), likely reflecting relatively poorer vascularity and thinner mucosa, three of our four cases involved the anterior maxilla.^1^ Dental extractions preceded MRONJ in two of our four cases. Extractions are implicated in up to 82% of MRONJ cases and markedly increase risk in oncology patients receiving intravenous bisphosphonates.^1^^,^^53^ However, evidence increasingly suggests that underlying dental infection or inflammation, rather than the extraction itself, may be the key precipitant, with MRONJ correlating more strongly with symptomatic or infected teeth and features such as purulent discharge.^39^^,^^49^^,^^54^^,^^55^^,^^56^ It has been proposed that local infections increase tissue acidity, enhancing bisphosphonate release and antiresorptive effects.^55^ Radiographic abnormalities (notably periapical osteosclerosis and apical lesions) predicted MRONJ, with higher risk when >1.1 abnormalities per tooth were present.^48^

Dental risk factors were prominent in this series. Three patients had periodontal disease (Cases 1, 2 and 4), three had heavily restored dentitions (Cases 1, 3 and 4) and one patient wore dentures (Case 2). Denture-related mucosal trauma has been reported in MRONJ. One study reported an approximately two-fold increased risk among denture wearers, while another implicated denture-pressure ulcers in over one-quarter of MRONJ cases.^57^^,^^58^ Periodontal inflammation and associated alveolar bone loss are consistently linked with MRONJ risk, with patients reported to be 2.75 times more likely to have periodontitis.^59^^,^^60^^,^^61^ In all four cases, ART was initiated without pre-treatment dental optimisation, which may have contributed to the burden of baseline oral disease at presentation. These observations support the emphasis placed on early dental screening and optimisation in patients commencing ART.

Prevention remains the most effective strategy. The MASCC/ISOO/ASCO clinical practice guideline recommends a pre-ART clinical and radiographic assessment, followed by a dental follow-up every 3–6 months once ART begins.^22^ For dental practitioners, MRONJ risk reduction includes treatment of periodontal disease, optimisation of oral hygiene, management of caries, adjustment of ill-fitting dentures, early management of mucosal trauma and addressing uncontrolled diabetes and tobacco use as early as possible.^22^^,^^62^ When invasive dentoalveolar procedures are unavoidable, careful case selection, atraumatic technique and close post-operative review to confirm complete mucosal healing are essential.

The primary aims of management in this case series were symptom relief, infection control and disease stabilisation. In accordance with MASCC/ISOO/ASCO and AAOMS guidance, initial management prioritised local measures, such as oral hygiene optimisation, chlorhexidine mouthrinses, local irrigation and removal of loose sequestrum, while reserving systemic antibiotics for clinically infected lesions.^1^^,^^22^ Imaging was based on clinical need. OPG radiography was used for initial assessment and follow-up, and cone beam computed tomography (CBCT) or CT was employed when delineation of three-dimensional extent was required for staging, to assess association to the maxillary sinus or mandibular canal and to support surgical planning.^63^

Systemic antibiotics were prescribed only when there were clinical features of secondary infection or as peri-operative adjuncts in infected cases, supporting antimicrobial stewardship.^22^ Empiric regimens for acute infective exacerbations were amoxicillin 500 mg TDS plus metronidazole 400 mg TDS for five days or augmentin 625 mg TDS for 5–10 days alongside local antimicrobial measures. In this series, doxycycline was used selectively as an adjunct for chronic, infected MRONJ. Doxycycline 100 mg was prescribed OD for 14–28 days, extending only if symptoms persisted and reviewing at follow-up to support antimicrobial stewardship. The aim was to reduce pain and soft-tissue symptoms and to optimise the local environment before and after debridement, rather than to use doxycycline as curative monotherapy for advanced disease. This reflects local unit practice and is supported by doxycycline's pharmacological profile. In tandem with its antimicrobial activity, doxycycline has anti-inflammatory, anti-collagenase and anti-osteoclastogenic effects and can penetrate bone well, achieving therapeutic concentrations with sustained activity against the polymicrobial flora typical of MRONJ lesions.^64^^,^^65^^,^^66^^,^^67^^,^^68^^,^^69^^,^^70^^,^^71^

Adjunctive tetracyclines have supportive evidence. A randomised controlled trial reported better symptom-free outcomes with surgical debridement plus topical tetracycline than surgery alone (90% versus 40% at three months).^72^ Studies suggest that local and systemic doxycycline may reduce inflammatory markers and support healing, with preclinical data indicating that doxycycline can enhance bone formation and modulate osteoclast activity.^73^^,^^74^^,^^75^ Tetracycline fluorescence has also been used in the literature to assist in intra-operative demarcation of viable bone, although this technique was not applied in the present series.^76^

Where possible, microbiological sampling (deep swab from exposed bone or a sinus tract and/or intra-operative tissue or bone specimen) was obtained with a specific request for *Actinomyces* culture, given its frequent association with MRONJ biofilms.^77^ Infectious diseases or microbiology consultation was not routinely sought for these four cases, but is considered for refractory infection, atypical organisms, immunocompromised patients or when prolonged courses are contemplated.

All four patients in this series ultimately required operative management because of persistent exposed bone with recurrent infection and/or progressive destruction despite conservative measures. Surgical decision-making was guided by symptom burden, progression and anatomical risk (for example, sinus or nasal floor proximity and mandibular fracture). Evidence suggests that advanced MRONJ responds poorly to conservative management and that surgical intervention achieves higher resolution rates in malignant cohorts, with reported healing rates of 88–97% in multiple myeloma.^78^^,^^79^^,^^80^^,^^81^^,^^82^^,^^83^ Earlier surgery may be beneficial, as lesions persisting beyond 12 months are associated with greater severity.^40^

Key surgical procedures in MRONJ management include complete removal of necrotic bone, smoothing of bony edges, mucosal closure and achieving an asymptomatic state within four weeks post-operatively.^78^^,^^79^ Debridement or sequestrectomy to healthy bleeding bone with primary closure where feasible was the principal approach, recognising that complete removal was not always possible in the maxilla without creating a clinically significant oro-nasal communication. Adjunctive local antimicrobial and antiseptic measures were used alongside surgical management. Intra-operative osteoconductive aminoglycoside calcium sulfate beads (Gentamicin, Vancomycin) can provide local antimicrobial delivery while filling surgical dead space. Whitehead's varnish was used post-operatively to reduce capillary oozing, alleviate pain and protect soft tissue or bony cavities from infection, thereby facilitating oral feeding and wound healing.^80^ All cases had severe MRONJ, with extensive maxillary oro-nasal communications and one pathological mandibular fracture. Staged management still achieved mucosal healing in three patients and stable symptom control in the fourth.

MRONJ prognosis is influenced by stage, with Stage 1 and Stage 2 lesions generally achieving better outcomes than Stage 3.^81^ Complications such as paraesthesia, fistulae and oro-antral communications are associated with poorer outcomes due to the need for more extensive intervention and prolonged healing.^82^ MRONJ site may also influence prognosis. Some studies report better outcomes in maxillary disease, including a five-fold improvement reported by Ruggiero and Kohn, with other studies noting that maxillary and posterior mandibular sites are predisposed to more severe presentations.^40^^,^^41^^,^^81^^,^^83^

The value of temporary interruption (‘drug holidays') in oncology-dose ART remains uncertain. AAOMS and MASCC/ISOO/ASCO guidance conclude that evidence is insufficient to support or refute routine discontinuation before dentoalveolar surgery or during MRONJ management, and decisions should therefore be individualised in conjunction with oncology teams, balancing potential oral benefit against the risk of SREs.^1^^,^^22^ In some cases, ART dose modifications may be considered.^84^ Recent evidence suggests that quarterly bisphosphonate infusions reduce MRONJ risk 7.75–8-fold compared to monthly dosing.^51^^,^^85^ ASCO found no difference in SREs between monthly and three-monthly 4 mg intravenous ZA in breast cancer patients with bone metastases.^86^ Pharmacokinetics influence the rationale for drug holidays. Bisphosphonates, despite 95% being excreted within six hours, have a skeletal half-life exceeding ten years, therefore maintaining antiresorptive effects long after discontinuation.^62^^,^^84^^,^^87^ In contrast, denosumab does not accumulate in bone, has a 26-day half-life, and may allow faster MRONJ resolution with drug holidays, although findings are inconsistent.^33^^,^^88^^,^^89^^,^^90^

Evidence on the timing of ART interruption is mixed. Some studies report that a ≥3-month interval from the last ART dose before dentoalveolar intervention is associated with reduced MRONJ risk in cancer patients and that cessation before MRONJ diagnosis or treatment may be linked to faster healing and symptom resolution.^52^^,^^82^^,^^91^ However, reported benefit appears to vary by management strategy, with conflicting findings between conservative and surgical cohorts.^92^^,^^93^

## Conclusion

Dental prevention, including pre-ART assessments, treatment and routine reviews, is crucial for reducing MRONJ risk and should be a core component of treatment protocols for patients with malignancy. Early recognition and referral to oral surgery are essential when managing MRONJ cases as surgical intervention is often necessary to achieve optimal outcomes. Referral to oral and maxillofacial surgery is recommended for extensive MRONJ cases, particularly those with a high risk of fracture or refractory to previous surgical interventions.
